# Understanding Social Media Addiction: A Deep Dive

**DOI:** 10.7759/cureus.72499

**Published:** 2024-10-27

**Authors:** Jashvini Amirthalingam, Anika Khera

**Affiliations:** 1 General Medicine, California Institute of Behavioral Neurosciences & Psychology, Fairfield, USA; 2 Medicine, Basis Education, Scottsdale, USA

**Keywords:** mental health, peer pressure, social media addiction, social media platform, teenagers

## Abstract

Social media, integral to contemporary life, offers significant connectivity and entertainment benefits. However, its pervasive use has given rise to social media addiction, particularly among teenagers, characterized by excessive screen time, compulsive checking, and detrimental effects on real-life relationships and responsibilities. This addiction is driven by a combination of psychological factors, such as low self-esteem and mental health issues, technological mechanisms like infinite scrolling and personalized notifications, and social influences, including peer pressure and exposure to idealized content. Effective management of social media addiction requires a multifaceted approach. Behavioral interventions, such as cognitive behavioral therapy (CBT) and mindfulness training, educational initiatives that raise awareness about addiction risks, and parental strategies involving boundaries and monitoring can collectively mitigate the negative impacts of social media. Implementing these strategies is crucial for fostering healthier online behaviors and improving overall well-being among teenagers.

## Introduction and background

Social media has become an integral component of everyday life. Instant entertainment, information, and connectivity make social media appealing. For some individuals, having access to these platforms transforms into an addiction. Social media addiction is characterized by excessive and compulsive usage that affects daily functioning and overall well-being. It encompasses more than just spending an excessive amount of time online; it involves compulsively checking for updates, feeling anxious when offline, and suffering from negative impacts on real-life relationships and responsibilities [1,2]. This addiction can manifest in several ways, such as avoiding personal and professional obligations and experiencing significant distress when unable to access any social media platforms. A broad array of stakeholders are influenced by and need to be involved in addressing this problem. 

## Review

Objectives

This review aims to summarize the prevalence and patterns of social media addiction among teenagers; identify psychological, social, and technological factors contributing to social media addiction; examine the psychological and social consequences of addiction; and review effective interventions and prevention strategies.

Prevalence and patterns of social media use

Prevalence

There is a range of prevalence rates of social media addiction among teenagers ranging from 5% to 20% [2,3]. These variations can depend on the specific criteria used to define addiction as well as the demographic characteristics of the research study. 

Patterns of Use

The most common ways that teenagers engage in social media platforms include activities such as endless scrolling, reacting to posts, and direct messaging in a digital environment. It is important to note that a study by Valkenburg et al. highlights that teenagers often spend an average of two to four hours per day engaged in various social media platforms, with many recognizing significant disruptions to their daily routines due to online interactions [4].

Psychological factors 

Self Esteem 

Self-esteem plays an important role in building self-identification during adolescence [5]. Teens go through significant transformations during this phase of growth as they discover who they are and how they communicate with others. Studies show that people with poor self-esteem regularly turn to social media for affirmation, which may heighten feelings of inferiority [5,6]. Social media is a double-edged instrument in the present digital world; it encourages comparison to well-chosen pictures and lifestyles while additionally making connections easier. Addiction to social media and dependence may result from this dynamic. According to Verduyn et al., the need for validation frequently manifests in the shape of increased online engagement and elevated anxiety when cut off, which feeds a vicious cycle of negative self-perceptions [6].

Mental Health 

Social media addiction is increasingly acknowledged as a significant factor impacting mental health [7]. Prolonged use of these platforms has been linked to heightened levels of anxiety and depression, particularly among adolescents and young adults [7,8]. Many individuals resort to social media as a coping mechanism to manage stress or loneliness. While these platforms can provide a sense of community, they often worsen feelings of inadequacy and isolation through constant comparisons to others' curated lives. Primack et al. discovered that individuals who spend excessive time on social media are more likely to experience symptoms of anxiety and depression [8].

In addition, the pressure to maintain an idealized online persona can lead to increased stress, resulting in a cycle of addiction as users seek validation through likes and comments [8,9]. Understanding the connection between social media usage and mental health is essential for developing healthier habits and coping strategies. Support from friends, family, and mental health professionals can assist individuals in effectively navigating these challenges.

Variable Reward System

Variable reward systems have been incorporated into social media platforms to increase engagement among users, particularly among teenagers. This system functions similarly to a gaming device since it establishes a dopamine-driven feedback loop whenever users are uncertain if they will be given likes, shares, or comments [3]. This cycle of incentives increases the probability that users would continue their online activities despite potential adverse consequences by encouraging obsessive checking and habitual interaction [3].

In particular, intermittent reinforcement may have significant effects. Teenagers who utilize social media show an intense desire to keep using it since they are uncertain if they will be approved or recognized. The reward pathways in the brain can be triggered by an expectation of achieving social approval [3].

Over time, increased participation generates a lucrative loop. This helps deepen persistent checking habits and continuous interactions. This could lead to a heightened rise in the amount of time spent online, exceeding any potential limits, like social isolation, depression, and anxiety [3]. It is important to have a long-term awareness of the internal functioning of these diverse incentive systems to understand how they impact user behavior.

Social factors

Peer Influence

One of the main reasons that adolescents rely on social media platforms is due to peer pressure. There is a strong desire for social acceptance and belonging that influences teenagers to participate in social media activities [10]. Through influence from their peers, many teenagers have to conform their interests and behaviors to those of their social circles. Valkenburg and Peter emphasize that this peer pressure is particularly evident in digital environments, where the pursuit of validation can lead to increased social media usage, often negatively impacting mental health [4].

Social Comparison

Apart from peer influence, social comparison is an important factor to take into account. People have many options to disclose personal information on digital platforms, including carefully chosen photos and idealized lives. Regular exposure to this kind of content fosters a comparison-friendly atmosphere. Chou et al. emphasize that this exposure can lead to unfavorable self-comparisons and feelings of inadequacy, which can cause problems including low self-esteem and social media addiction [11].

Peer pressure and social comparison can work together to create a vicious cycle whereby increased social media use is motivated by the need for approval and the fear of falling short of peers. Teenagers' self-acceptance and the development of healthy online behaviors depend on an understanding of these relationships. Promoting candid conversations regarding the effects of social media can enable teenagers to deal with these issues more skillfully.

Technological factors 

To really appreciate the influence of social media on teenagers' lives, one must comprehend how they use it. These platforms are especially interesting to younger users because of a number of important design elements that greatly improve user engagement.

Infinite Scrolling

The infinite scroll feature is one of the most important design components. This feature makes it possible for users to explore content constantly and uninterruptedly, resulting in a smooth experience that promotes extended use. Infinite scrolling makes it simple for users to lose track of time and engage in prolonged platform sessions by doing away with natural stopping points like page breaks [1,12].

Algorithm-Driven Content

Another important component is algorithm-driven content, which personalizes the posts that users view based on their previous interactions and preferences. Adolescents are more likely to see posts that are relevant to their preferences because of this customization, which also makes the material displayed more relevant. Studies indicate that this focused scheme not only enhances user participation but also builds a stronger bond with the platform [13].

Personalized Notifications

Customized alerts are essential for preserving user interest. Users are prompted to check their profiles often by the thrill and sense of urgency created by alerts about new likes, comments, or messages. Teenagers find it challenging to resist the impulse to log in and stay connected because of this continuous barrage of notifications, which fosters habitual use [14].

Psychological consequences

Mental Health Impacts

Teenagers who use social media regularly are more inclined to struggle with anxiety, depression, and a general decline in life satisfaction, according to multiple research studies. The long-term use of social media could negatively impact sleep quality, which exacerbates mental health problems, according to studies [7,15,16]. Adolescent eating disorders and appearance-related anxiety may worsen as an effect of the emphasis on ideals of beauty that permeate social media culture [7,17,18]. This pressure to live up to unrealistic expectations of beauty can lead to serious mental health problems and an adverse self-image [18].

Eating Disorders 

Growing rates of eating disorders, particularly bulimia, are a serious result of this phenomenon [19]. Binge eating, followed by purging, is a common method of coping used by individuals who have issues with their bodies, and it's frequently affected by the pressures they feel from social media [19,20]. In conjunction with establishing supportive instances that allow individuals to develop healthier self-images, promoting awareness of media and encouraging realistic body representations can help mitigate these consequences [21].

Cognitive Effects

Social media has a detrimental impact on cognitive function in addition to its impact on mental health. The excessive use of social media has been shown to negatively impact cognitive function and reduce attention span. Social media platforms' constant digital stimulation encourages attention to be diverted and affects the ability to focus on tasks for an extended amount of time [22]. Both educational achievement and the ability to engage deeply and meaningfully with the subject matter might be impacted by this brief attention span.

Social media's cumulative effects on mental health and cognitive function highlight how essential it is to raise awareness of these problems. Encouraging healthier social media behaviors can lessen these dangers and support teenagers in developing healthier connections with technology.

Social consequences

Relationships

Since social media platforms render it convenient to connect with people instantly, relationship preferences may shift drastically. Family ties might deteriorate as a result of these platforms, even while they provide opportunities for more social interaction. Because people commonly value online interaction over in-person interactions, research suggests that extensive social media use may result in lower-quality face-to-face encounters. This dynamic has the potential to negatively impact emotional well-being by diminishing familial ties and hindering the establishment of meaningful connections [4,23]. Furthermore, the instantaneity of digital communication might give rise to miscommunications and disputes, further straining interpersonal bonds.

Academic Performance

The harmful impact that social media has on student performance is another significant concern for parents. According to research, social media addiction can often result in distraction from learning responsibilities, which has an adverse effect on students' performance in educational settings, particularly among adolescents. Lower grades and diminished academic motivation could result from social networking's appeal and periodic updates, which can cause one to find themselves sidetracked from their educational pursuits [24]. This sort of distraction is especially harmful when learning is at its most vital, and success is contingent upon commitment and focus.

All things taken into account, social media helps individuals connect and share information; however, it also poses challenges that may negatively impact children's academic achievement and the quality of their interpersonal relationships. It is essential to recognize these potential downsides in order to nurture more balanced lifestyle practices and healthier electronic media habits.

Interventions and prevention strategies

Cognitive Behavioral Therapy (CBT)

One effective intervention for addressing social media addiction is cognitive behavioral therapy (CBT). This therapeutic approach has demonstrated significant benefits for teenagers struggling with excessive social media use. CBT works by helping individuals identify and alter unhealthy thought patterns and behaviors associated with their online activities. By fostering more adaptive thinking, CBT enables teenagers to recognize triggers for their social media use and develop healthier coping strategies [25]. Through this process, they can learn to manage their impulses and make more conscious choices regarding their digital interactions, ultimately reducing the negative impacts of social media on their lives.

Mindfulness and Self-Regulation

In addition to CBT, techniques such as mindfulness training and self-regulation strategies have also proven effective in managing social media addiction. Mindfulness practices encourage individuals to become more aware of their thoughts, feelings, and behaviors in the present moment. This heightened awareness can help teenagers recognize when they are engaging in compulsive social media use and allow them to pause and reflect on their choices [26]. Self-regulation strategies, including setting specific limits on social media use and creating designated "offline" times, further support adolescents in developing a balanced relationship with technology. Together, these approaches empower teenagers to take control of their social media habits and promote overall well-being.

Educational Programs

Raising awareness via campaigns is essential to combating social media addiction, particularly among adolescents. Education is crucial for such initiatives since it allows youth to recognize the risks and consequences of excessive social media use. Teens can learn how social media could influence their relationships, mental health, and academic performance through awareness-raising programs.

Studies highlight how important it is that we put in place educational initiatives that highlight the potential negative effects of social media, like melancholy, anxiety, and a decline in life satisfaction [27]. Such efforts have the ability to boost resilience while encouraging more balanced interactions with social networking sites by disseminating information on appropriate usage patterns and promoting critical thinking within online activities.

Awareness campaigns may also involve presentations at schools, workshops, and online resources that motivate teenagers to engage in conversation about their usage of social media platforms. By facilitating open conversations about individual struggles and experiences, these types of initiatives can de-stigmatize the widespread issue of social media dependence while developing an environment that encourages change.

Parental involvement

Guidance and Monitoring 

Effective interventions to combat social media addiction often involve active participation from parents and caregivers. Encouraging them to set boundaries and monitor their teenagers' social media usage can significantly reduce the risks of addiction. By establishing guidelines for acceptable online behavior, parents can help teens develop a healthier relationship with technology [28]. This proactive approach can also create opportunities for open discussions about the potential dangers of excessive use and the importance of balance.

Setting Time Limits

One practical strategy for managing excessive social media usage is setting time limits. Many social media platforms now offer integrated features that allow users to track and limit their daily usage, helping to instill more mindful consumption habits. Additionally, third-party applications are available to enforce screen time restrictions, promoting healthier online activities [22]. These tools not only empower individuals to regain control over their social media habits but also decrease the likelihood of developing addictive behaviors. By fostering awareness of how much time is spent online, users can make informed choices about their engagement with social media.

Facilitating Open Communication

To cultivate better social media routines, it's crucial to not only set limits but also promote candid conversations about actions online and their repercussions. Adolescents may reflect on their experiences, evaluate any negative effects, and develop their ability to think critically regarding their online behaviors by receiving encouragement to have truthful talks about their utilization of social media [29]. In addition to improving relationships between parents and children, this dialogue gives individuals the skills they require to navigate the world of technology with greater caution.

Discussion

Summary of Findings 

Critical considerations concerning the relationship between technology, mental health, and social standards are brought up by the growing problem of teen social media addiction. Social media platforms are meant to encourage connection, but they can also create circumstances that promote obsessive habits, especially in young people who are more vulnerable. The psychological elements, such as poor confidence and mental health issues, highlight the necessity to understand more about how social media can be a cause of anxiety as well as a coping method. Many youngsters find that their feelings of inadequacy are momentarily alleviated by the validation they receive from likes and comments. But frequently, this validation is ephemeral, creating a vicious cycle of dependency that can worsen pre-existing mental health conditions.

Several factors contribute to social media addiction among teenagers. Psychologically, low self-esteem and mental health issues like anxiety and depression can drive excessive use, as social media offers a coping mechanism and validation. Socially, peer pressure and exposure to idealized online images exacerbate addiction by fostering negative self-comparisons and a desire for social acceptance. From a technological point of view, social media engagement algorithms are becoming more complex and designed to maintain users' attention for as long as possible. Features like endless scrolling are implemented, which increases the risk of addiction and normalizes prolonged usage. As a result, people find it more difficult to notice when their online behavior becomes problematic. 

Addressing social media addiction involves a multi-faceted approach. Behavioral interventions like cognitive behavioral therapy (CBT) and mindfulness training have shown promise in managing addiction. Educational programs that raise awareness about the risks and parental strategies, such as setting boundaries and monitoring usage, are also effective. By integrating these strategies, it is possible to mitigate the negative effects of social media addiction and promote healthier online behaviors among teenagers.

Limitations

There is variability in study methodologies and definitions of addiction. Many studies also lack longitudinal data. Future research should focus on longitudinal studies to understand the long-term impacts of social media addiction and explore new interventions, including the role of emerging technologies to help compact addiction to social media platforms. An increase in the widespread use of subjective and objective tools to help measure social media addiction will also be helpful for parents, teachers, and clinicians.

## Conclusions

Social media addiction among teenagers is a growing concern with far-reaching effects on mental health, social relationships, and academic performance. Addressing this issue requires a multidisciplinary approach with the need for collaborative stakeholder efforts to lead to increased use of behavioral therapies, educational initiatives, and parental involvement to address this issue. Promoting awareness through education, creating more tools for both subjective and objective measurements of addiction, and fostering open communications about the topic can help reduce the negative impacts on teenagers. 
